# Distinct Roles of Histone H3 and H2A Tails in Nucleosome Stability

**DOI:** 10.1038/srep31437

**Published:** 2016-08-16

**Authors:** Zhenhai Li, Hidetoshi Kono

**Affiliations:** 1 Molecular Modeling and Simulation Group, National Institutes for Quantum and Radiological Science and Technology, 8-1-7 Umemidai, Kizugawa, Kyoto 619-0215, Japan

## Abstract

Nucleosome breathing potentially increases the DNA exposure, which in turn recruits DNA-binding protein and regulates gene transcription. Numerous studies have shown the critical roles of N-terminal tails of histones H3 and H4 in gene expression; however, few studies have focused on the H2A C-terminal tail. Here we present thorough computational studies on a single nucleosome particle showing the linker DNA closing and opening, which is thought to be nucleosome breathing. With our simulation, the H2A C-terminal and H3 N-terminal tails were found to modulate the nucleosome conformation differently. The H2A C-terminal tail regulates nucleosome conformation by binding to linker DNA at different locations, whereas the H3 N-terminal tail regulates linker DNA by binding to it in different patterns. Further MD simulation on tail truncated structures corroborates this analysis. These findings replenish our understanding of the histone tail regulation mechanism on atomic level.

In eukaryotes, genetic material, i.e., genomic DNA, is stored in the nucleus as a protein–DNA complex, chromatin. The fundamental structural unit is the nucleosome, which is successively connected to other nucleosomes by linker DNA and folds into chromatin. The nucleosome consists of eight histone proteins and DNA. These eight histone proteins include two copies of each of H3, H4, H2A, and H2B histone proteins. H3 and H4 histone proteins form a dimer and two of these dimers form a tetramer. H2A and H2B histone proteins form a dimer, and two H2A–H2B dimers symmetrically attach on opposite sides of the H3–H4 tetramer, eventually forming the histone core octamer. The DNA wraps around the histone octamer in a left-handed superhelical turn with approximately 150 bp^1^,^2^. In addition to these structured compartments, each histone protein has a tail on their N-terminal end that is a highly intrinsically disordered region^3^; besides, each H2A histone protein has an additional unstructured C-terminal tail (H2ACtT). The positively charged histone tails favorably interact with nucleosomal DNA^4^, linker DNA^1^,^5^,^6^, and acidic patches on the histone core^7^,^8^.

Among all histone tails, the H3 N-terminal tail (H3NtT) is the longest. It protrudes from the nucleosome core through the channel formed by the minor grooves of DNA strands at the entry/exit region. Because of numerous post-translational modification sites on H3NtT, it has been extensively studied. H3 and H4 N-terminal tails mediate the packing of the nucleosome into a higher-order structure through their binding to DNA or the acidic patch on neighboring nucleosomes^9^,^10^,^11^. At the single nucleosome level, removal of the H3 and H4 N-terminal tails destabilizes the nucleosome structure and increases DNA exposure^12^,^13^,^14^,^15^,^16^. The acetylation of H3 enhances DNA unwrapping^17^. Deletion or alanine mutation of H3NtT promotes nucleosome sliding along the DNA^15^. As the only C-terminal tail, H2ACtT is critical to chromatin structure and dynamics^18^. Biochemical study has shown that H2ACtT contacts nucleosomal DNA at the dyad axis in an isolated nucleosome^19^. H2ACtT recruits linker histone H1 to the nucleosome and stabilizes it^18^. In linker histone-depleted chromatin, it interacts with linker DNA^7^. Deletion of H2ACtT results in an increase of H2A mobility and a decrease of nucleosome stability^18^.

However, because of the resolution limit of most experimental techniques, it is difficult to investigate the mechanism of histone tail regulation at the single-molecule level. Thus, to better understand nucleosome dynamics, all-atom molecular dynamics (MD) simulations were performed on the histone core, DNA strands^20^,^21^, histone tails^22^,^23^, DNA strands and histone tails fragment^24^,^25^,^26^, single nucleosome particle^27^,^28^,^29^,^30^, and two stacked nucleosome particles^31^. Using simulations on DNA strands only and DNA wrapped around the histone core, researchers studied protein–DNA interactions, and showed how these interactions influence DNA’s mechanical properties, structure, and dynamics^20^,^21^,^27^,^29^,^30^. Using a large batch of simulations on nucleosomes with distinct DNA sequences, researchers investigated how the DNA sequence affects nucleosome dynamics^32^. Simulations on the histone tail revealed a single dominant binding configuration of H4 and H2A histone N-terminal tails on the nucleosome core, and multiple binding configurations of H3 and H2B. The interaction between the histone tails and nucleosome core tends to destabilize the secondary structure of the histone tails^22^,^23^. In MD simulation and molecular docking calculation, H4 N-terminal tails were found to mediate DNA-DNA strand attraction. Furthermore acetylation and mutation on lysine or arginine in H4 tail have great impact on structure of DNA strands and H4 tail complex^24^,^25^,^26^. Study of stacked nucleosome particles further suggested H4 histone tails play important role in inter-nucleosome interaction by regulating nucleosomal DNA interaction. Moreover H2A N-terminal tails were found to be critical to inter-nucleosome interaction as well^31^. Steering MD simulation on the unwrapping of DNA from the nucleosome revealed how the histone core and tail stabilize the nucleosome structure, and what specific role each histone core and tail play in stabilizing the nucleosome^33^. A controversial function of H3NtT was found in the multiscale simulation on unwrapping nucleosome, i.e., stabilization of the opening conformation of nucleosome^34^. However, these simulations showed that the nucleosome is relatively stable within a 100-ns timescale. Recently two research groups showed extended simulation^29^,^30^. In 250-ns long MD simulation, Bowerman and Wereszczynski focused on effects of H2A variants on nucleosome dynamics^29^. With 1-us long MD simulation, Shaytan *et al*. investigated unconstrained nucleosome, and observed nucleosome breathing^30^.

Here, we present our MD simulation of a nucleosome particle with 167 bp of DNA wrapped around the histone core and all histone tails (see details in Methods). To obtain as many conformational changes as possible, we performed 10 independent, 100-ns long, all-atom MD simulations at a relatively high temperature (353 K) to accelerate the dynamics. In these simulations, we successfully observed the nucleosome structural changes, in which the linker DNA ends either approaching or departing each other. We termed these two opposite processes as nucleosome closing or opening. In the structural changes, the end-to-end distance varies from ~0 to ~8 nm, which covers the range of nucleosome breathing. On closely examining the closing and opening processes, we found that H2ACtT and H3NtT play apivotal role in regulating linker DNA dynamics and nucleosome structures. To confirm these findings, we performed 26 additional independent simulations on the nucleosome particles with either H2ACtT or H3NtT truncated (listed in Table 1). The results revealed distinct regulatory mechanisms for H2ACtT and H3NtT. Moreover, the stability of the closed conformation was tested by additional MD simulations (Table 1). In the two 100-ns long simulations, we did not observe significant conformational change for an intact nucleosome. However, different reopening behaviors were found in the nucleosome with H2ACtT, H3NtT, or both tails truncated. In the case with both tails truncated, the nucleosome, which was initially in the closed conformation, rapidly transitioned into the open conformation.

## Results

**Figure 1:**
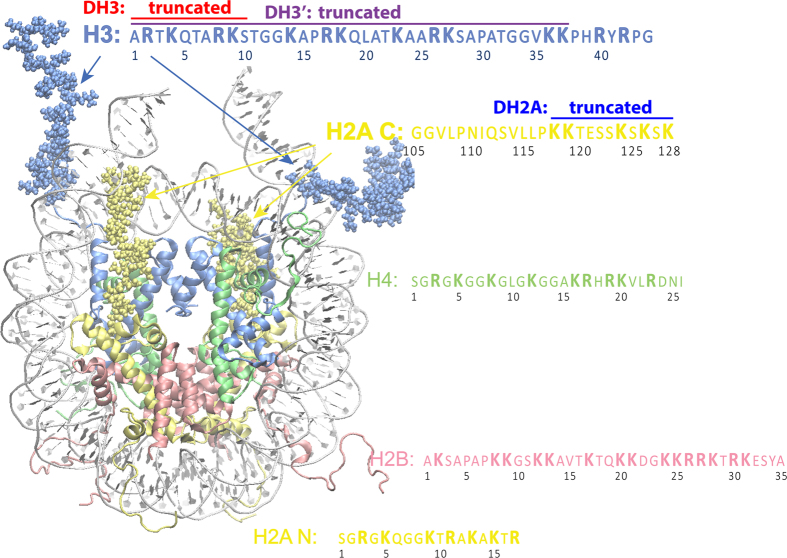
Simulated nucleosome structure and histone tails. DNA and histone proteins are labeled with different colors. DNA contains 167 bp. Histone protein contains all of the histone tails, as listed in the figure. Figure was created with VMD^76^.

In this study, we used a nucleosome with 167 bp of DNA, which included 147 bp of nucleosomal DNA with an additional 10 bp of linker DNA on each end, as shown in Fig. 1 because previous studies have suggested that H3NtT and H2ACtT have the potential to interact with linker DNA^1^,^5^,^6^. All simulations performed in this study are summarized in Table 1.

### Molecular dynamics simulation of a single nucleosome particle

**Figure 2:**
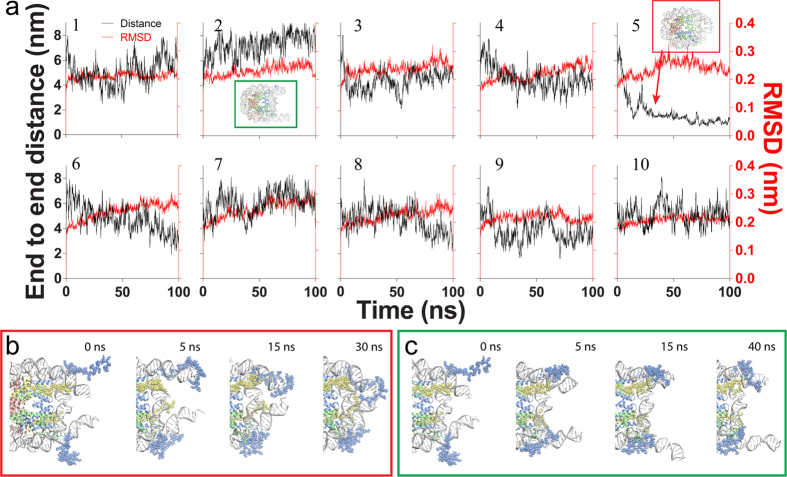
Fluctuations of intact nucleosomes from ten independent molecular dynamic simulations. (**a**) rmsd for histone core and a 127-bp segment of nucleosomal DNA of heavy atoms (red) and linker DNA end-to-end distance (black). The second and fifth simulations exhibit significantly different end-to-end distances compared with the other simulations. The insets in red and green boxes show two simulations with closed and open conformations, respectively. (**b,c**) Show intermediate snapshots of the fifth and second simulations, which transitioned to a closed or open conformation, respectively.

Ten independent simulations were performed based on this structure with different initial velocities. Although the histone fold domains and nucleosomal DNA showed greater variations in root mean square deviations (rmsds) of heavy atoms of nucleosome at 353 K than at 300 K, as expected (Fig. 2a and Supplementary Fig. S1), the rmsds reached equilibrium at least within 20 ns (red curve in Fig. 2a). This is consistent with previous studies, which suggested that the histone core proteins are relatively stable^27^,^35^,^36^. However, the histone tails adopted different conformations and bound to DNA at different locations (Supplementary Fig. S2), which was also observed in a previous study^30^,^33^. The positively charged amino acids, lysine and arginine, are prevailing in the interactions between tails and DNA strands (Supplementary Fig. S3), suggesting the electrostatic interaction plays critical role in the interactions. Besides, the linker DNA adopted different conformations among these simulations. The distance between two ends of the linker DNA varied from ~0 to ~8 nm (Fig. 2a black curves, Supplementary Fig. S4), covering the range of linker DNA distances shown in previous fluorescence resonance energy transfer (FRET) studies on nucleosome breathing^16^,^37^. According to the crystal structure of the nucleosome, the distance between two ends (*D*) is approximately 4 nm^38^. In our simulation, two out of 10 runs showed significant deviations from the initial structure, which had *D* of 7–8 nm and 1–2 nm (Supplementary Videos 1&2, and the second and fifth simulations in Fig. 2a, respectively). We named these two states as open and closed conformations accordingly. All remaining structures were referred to as an intermediate state.

Apparently, the closed and open states correspond to two opposite states in breathing. The former one had two linker DNAs closely wrapped around the histone core whereas the latter one had both linker DNAs in a relatively open conformation with the nucleosomal DNA near entry/exit and inner turn of DNA at the dyad exposed to the environment. Since the opening and closing of linker DNAs are considered to be closely related with the activation and repression of gene transcription, respectively, we focused on these two simulations to investigate the mechanism of linker DNA closing and opening.

Figure 2b,c show the closing and opening processes in the time course of simulation, respectively. In the closing process, one of H3NtTs first bound to the end of the linker DNA on the same side at 5 ns; next, the linker DNA swung in the solution with H3NtT attached to it, and the H3NtT was brought close to the linker DNA on the other side during the swinging; the H3NtT occasionally made contact with the linker DNA on the other side (as shown at 15 ns in Fig. 2b); and finally, the H3NtT mediated interactions between linker DNAs and stabilized the nucleosome in a closed conformation. Meanwhile, we observed the jumping of H2ACtT between nucleosomal and linker DNAs. Initially, H2ACtT interacted with the minor groove of linker DNA proximal to the entry/exit region. As the swing of DNA repeatedly brought H2ACtT to the inner turn of DNA at the dyad, H2ACtT occasionally jumped between the linker DNA and nucleosomal DNA at the dyad position (Supplementary Video 3). Because of the positively charged Lys residues in H2ACtT, eventually the C-terminal tail formed stable interactions with both linker DNA and the inner turn of DNA in the vicinity of the dyad. In contrast to the closing process, we did not observe either H2ACtT binding to the distal end of linker DNA or H3NtT binding to both linker DNAs in the opening process.

### Location and Role of the H2A C-terminal tail

**Figure 3:**
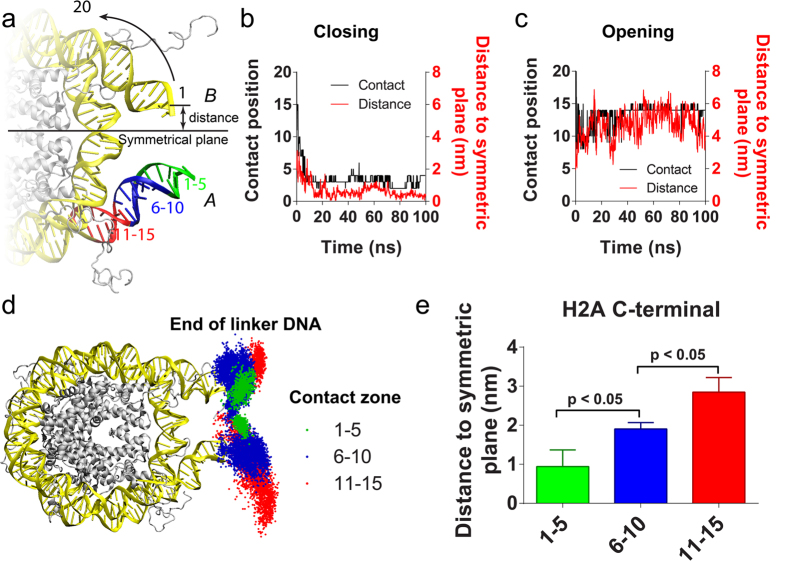
The correlation between the DNA position that H2ACtT interacted with and linker DNA motility. (**a**) Schematic of DNA base pair labeling rule and three contact regions on DNA. (**b,c**) Show the DNA positions that H2ACtT contacted with (black curve) and the distance from the linker DNA end to the symmetric plane (red curve), along with the simulation time in the closed and open simulations. (**d**) The distribution of linker DNA end locations sampled in all 10 simulations. Each point in the figure represents a location of the linker DNA end. The points were classified into three groups according to the regions where H2ACtT was in contact with DNA. When H2ACtT contacts DNA at 1–5 bp from the end, the points were colored in green; for 6–10 bp from the end, the points were colored in blue; for 11–15 bp from the end, the points were colored in red. (**e**) The average distance from the linker DNA end to the symmetric plane of the above three groups. Standard errors in the figure are calculated from the independent distances obtained from different simulation runs. The P-value is calculated by two-tailed t-test.

To unravel how H2ACtT interact with, and in turn regulate linker DNA, we first examined how distally H2ACtT can reach the linker DNA. The DNA base pairs were numbered, beginning from 1 at the most distal base pair until 20 at the 20^th^ base pair from the DNA distal end, as shown in Fig. 3a. The most distal DNA base pairs that made contact with H2ACtT were marked and its number was recorded as the contact position. Thus, a smaller contact position number indicates that H2ACtT reaches the more distal end of the linker DNA (see details in Methods). The same analysis was applied to both DNA ends and corresponding H2ACtTs. We distinguish two DNA ends, H2ACtTs and H3NtTs by giving an index of “*A*” or “*B*” (Fig. 3a). Meanwhile we also analyzed the distance from the corresponding linker DNA end to the symmetric plane (which was defined as a plane that passes through dyad axis and DNA superhelix axis) of the nucleosome. The black curves in Fig. 3b and c show the variations in the H2ACtT-*B* contact position on DNA-*B* during the simulations in the closing and opening processes, respectively. While the red curves show the variations in DNA end-symmetric plane distance on the same DNA end. The contact position and the distance simultaneously changed in both cases. Similarly, simultaneous changes of H2ACtT-DNA contact positions and the distances of the DNA end to the symmetric plane were found in our other simulation runs (green and red curves in Supplementary Fig. S4).

According to the DNA end-to-end distance and rmsd (Fig. 2a), the nucleosome core structure reached equilibrium within 20 ns and the distance remained relatively stable thereafter. Thus, we used the simulation trajectories after 20 ns for the following analysis. We found that H2ACtT mostly bound to DNA at contact positions within the range 1–15. The contact position distribution showed three peaks at 3, 8, and 14 on one side of linker DNA, and at 6 on the other side (Supplementary Fig. S5). Based on this observation, we split the DNA into three zones: 1–5, 6–10, and 11–15 (Fig. 3a). Conformations obtained by all ten simulations were divided into three groups according to the zones to which H2ACtT bound, and accordingly the positions of linker DNA were plotted with different colors in Fig. 3d after structural alignment to the initial conformation. Obviously, the movement range of the DNA end was greatly suppressed by H2ACtT being in contact with the distal linker DNA. The average distance from the DNA end to the symmetric plane was also calculated for each group and was plotted in Fig. 3e. Because the nucleosome is structurally pseudo-symmetric, two H2ACtTs regulate linker DNAs in the same manner. Both DNA ends and H2ACtTs are considered together in Fig. 3e. The figure shows the distance is significantly correlated with the DNA zones H2ACtT touches. Apart from the distance, the fluctuation was analyzed. We used the standard deviation of the distance within 1 ns to characterize this fluctuation. Similarly, the fluctuation was reduced with H2ACtT being in contact with the distal end (Supplementary Fig. S6).

### Location and Role of the H3 N-terminal tail

In contrast to H2ACtTs, which bound to the linker DNAs only from the inside (histone core side), H3NtTs showed various patterns of interaction with linker DNA. In fact, we did not observe a clear correlation between the contact position and the distance between the DNA end and the symmetrical plane (Supplementary Figs S4 and S6), unlike the case for H2ACtT.

**Figure 4:**
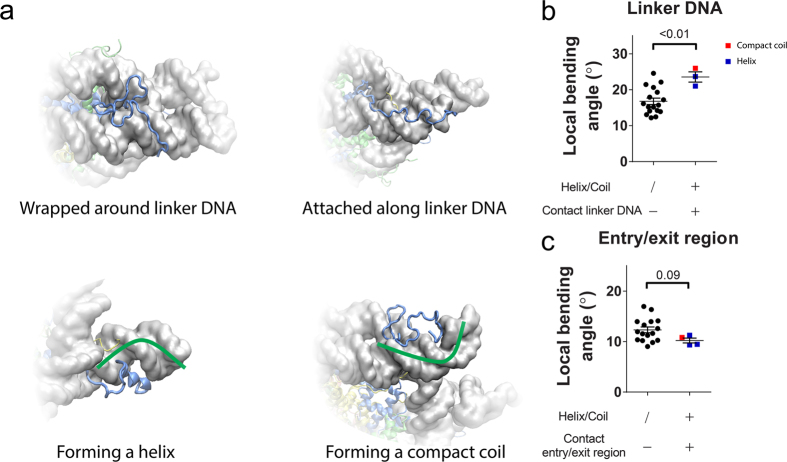
Representative interaction patterns of H3NtT to DNA and DNA bending. (**a**) H3NtT is wrapped around DNA along its minor groove (top left). H3NtT is attached to DNA and arranged parallel to it (top right). H3NtT forms an α-helix on the external side of DNA (bottom left). H3NtT is curled up on the external side of DNA (bottom right). (**b**) DNA local bending at linker DNA. (**c)** DNA local bending at entry/exit region. Standard errors in the figures are calculated from the independent distances obtained from different simulation runs. The P-value is calculated by two-tailed t-test.

Then, we focused on the correlation between the linker DNA conformation and binding patterns of H3NtT. We grouped all of the patterns into four types as follows (Fig. 4a): (i) the H3 tail attaches to DNA and extends parallel to the DNA helical axis; (ii) the H3 tail wraps around DNA along either its major or its minor groove; (iii) the H3 tail forms an α-helix which lasts over a half of the simulation time and resides in either the major or the minor groove of DNA (Supplementary Fig. S7); and (iv) the H3 tail forms a compact coil of which average radius gyration is significantly smaller (with 95% confidence interval) than that of the type iii (Supplementary Fig. S7). Because of the long length of H3NtT, the binding patterns are not exclusive to each other. The complicated binding patterns of H3NtT result in complicated regulation of the linker DNA conformation.

The simulation trajectories showed that local curvature of DNA was affected when H3NtT formed an α-helix or a compact coil conformation. When H3NtT with helix or compact coil conformation contacted to the linker DNA, it appeared to force the linker DNA to bend (Fig. 4a lower two panels). At entry/exit region, the DNA strands are attracted to histone fold domains, which tend to induce DNA bending around the histone core. When the histone tail with a helix or compact coil contacted the entry/exit region from outside, the contact counterbalanced the interaction between the histone fold and DNA, thus straightening DNA. In another word, it reduced the local bending angle. Again because the histone tail usually attached to the linker DNA from outside, the bending of linker DNA and the straightening of DNA at entry/exit region may result in exposing the nucleosomal DNA (Supplementary Fig. S7). To quantitatively investigate the effect from H3NtT, we analyzed the secondary structure of both H3NtTs in all ten simulations (see details in Methods, and Supplementary Fig. S7). We regarded a stable helix conformation when it lasted over half of the simulation time. The radius of gyration (RG) of the first 37 amino acid of H3NtT was calculated as well (Supplementary Fig. S7). The tail with compact coil was defined as one with RG significantly less than average RG of tails with helix (see details in Methods, and Supplementary Fig. S7). These stable helices and a compact coil were further grouped into three cases: (i) contacting with linker DNA, (ii) contacting with entry/exit region, (iii) not contacting with DNA. Meanwhile the local bending angle of linker DNA and entry/exit region were analyzed (see details in Methods) as shown in Supplementary Fig. S7. Similarly, two H3NtTs regulate linker DNA in the same manner, thus we consider these analysis of both ends in all ten simulations together. As shown in Fig. 4b,c, the maximum and minimum of the bending angle of both linker DNA and both entry/exit region in all ten simulations were respectively plotted according to the group to which corresponding H3NtT belonged. Taken together, we conclude that condensation of H3NtT (adopting compact conformation or helix) is likely to induce the DNA bending in the direction of DNA dissociation.

In addition, H3NtT has the possibility of reaching the linker DNA on the other side in the closed conformation because of its long length. In this case, H3NtT resides between the linker DNAs and stabilizes the closed conformation by forming several hydrogen bonds with both linker DNAs (Supplementary Fig. S8). The positively charged Lys and Arg residues in the tail stably interacted with both linker DNAs, forming a DNA–tail–DNA bridge, which stabilized the nucleosome in the closed conformation, as shown in Fig. 2b and Supplementary Fig. S8.

### Distinct roles of H2ACtT and H3NtT in regulating opening and closing

The linker DNA position was highly correlated with the position at which H2ACtT contacted DNA (Fig. 3), but this was not the case for H3NtT (Supplementary Figs S5 and S6). H3NtT bound to linker DNA in various patterns (Fig. 4). These various binding patterns may result in enhancing or suppressing the DNA opening. Therefore, we expected that the truncation of H2ACtT (DH2A) would greatly affect linker DNA fluctuation, but not would the truncation of H3NtT.

**Figure 5:**
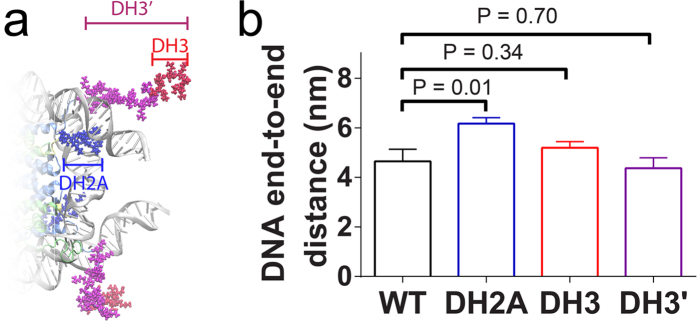
Impact of tail truncation. (**a**) Truncated sites of H3NtT (DH3 and DH3′) and H2ACtT (DH2) tails are shown in red, magenta, and blue respectively. In each case, both copies of histone tails are truncated. (**b**) A comparison of the end-to-end distance between the intact nucleosome and H2ACtT or H3NtT truncation nucleosomes. Standard errors in the figure are calculated from the independent distances obtained from different simulation runs. The P-value is calculated by two-tailed t-test.

To validate this hypothesis, we performed additional MD simulations for nucleosome particles with H2ACtT (11 amino acids) or H3NtT (10 or 37 amino acids) truncated (Figs 1 and 5a). For each of the tail truncation systems, ten (or six for 37 amino acids deletion of H3NtT, denoted by DH3′) independent 60 ns-long MD simulations were performed. The mean value of the linker DNA end-to-end distance was calculated using the last 40-ns trajectory for each simulation. The average end-to-end distance was eventually obtained as the average of mean values of ten simulations. As we expected, the average distance was significantly increased in H2ACtT truncation system compared with that of intact nucleosome system. However, the average distance was not affected by H3NtT truncation of either 10 or 37 amino acids (Fig. 5b). These results support our expectation that H2ACtT has more direct impact on the linker DNA fluctuation than H3NtT.

### Nucleosome opening from the closed conformation

H2ACtT and H3NtT have been shown to play critical roles during the opening and closing processes. However, the roles that these tails play in the closed conformation were yet unclear. To test whether the truncation of H2ACtT or H3NtT destabilizes the closed nucleosome, additional simulations starting with a closed conformation were performed on intact nucleosome, nucleosome with H2ACtT truncated, nucleosome with H3NtT truncated, and nucleosome with both tails truncated. In each system, two independent, 100-ns-long simulations were conducted. The minimal linker DNA end-to-end distance was monitored during these simulations. From these simulations, we observed three nucleosome states: (i) a closed state with the minimal DNA end-to-end distance of <0.5 nm; (ii) a partially closed state with the minimal DNA end-to-end distance of approximately 1 nm, and (iii) an intermediate state with the minimal DNA end-to-end distance of approximately 3 nm. In the cases of an intact nucleosome and a nucleosome with H3NtT truncated, the distance varied between 0 and 1 nm, indicating that the nucleosome fluctuated between a closed and a partially closed state, and the proportion adopting the partially closed state slightly increased for the nucleosome with H3NtT truncated compared with the intact nucleosome. In the case of the nucleosome with H2ACtT truncated, it fluctuated between the closed and intermediate states. The closed or partially closed states were exhibited in over 80% of the simulation time. In the case with the truncation of both histone tails, the nucleosome rapidly transitioned to the intermediate state, in which it then remained relatively stable.

**Figure 6:**
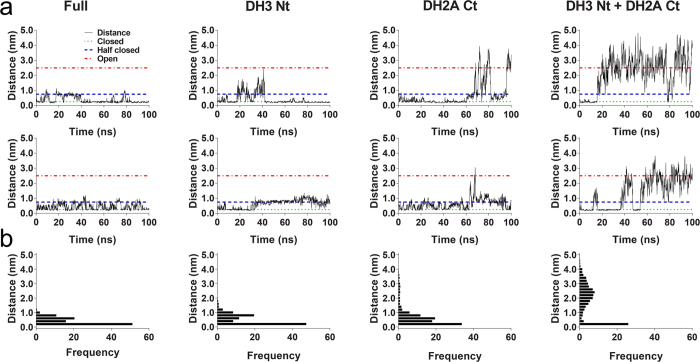
The minimal DNA distance. Simulations started with a closed conformation for the intact system, and those with truncation of H3NtT, H2ACtT or both tails. (**a**) The minimal DNA end-to-end distance over time for the different systems in different columns, as labeled on the top. The upper row and lower row are for two independent simulations for each system. (**b**) Distribution of the minimal end-to-end distances obtained by two independent runs for each of different systems.

For the intact nucleosome system, we did not observe opening of the nucleosome within 100 ns in two independent simulations (Fig. 6). The structure showed very limited changes in rmsd (Supplementary Fig. S9). Taken together, we consider that the process of opening of an intact nucleosome occurs slower than can be detected using the current all-atom simulation capability. In fact, the opening kinetics measured by FRET is slower than that for closing by a factor of at least five^39^.

## Discussion

We have carried out the total of 3.36 μs long MD simulations on nucleosomes to analyze the behavior of nucleosomal DNA associated with histone tails and found distinct roles of H3NtT and H2AcT for nucleosome stability. It is noteworthy that our MD simulations were performed under elevated temperature, which accelerates kinetics. This, on one hand, makes sampling of the histone tails positions and movements quite extensive, “effective” simulation time is much longer than formal total of 3.36 μs, but, on the other hand, make it difficult to compare the opening and closing rates with experiment results. However, a previous study suggested, within 100 ns, histone tail-DNA binding barely change^30^,^33^. With the temperature elevated, we still barely see the tail-DNA binding rearrangement (Supplementary Fig. S10), thus to further extend our sampling of the tail-DNA binding conformation, we performed independent simulations in the same conditions. These independent simulations exhibit that the interaction between histone tail and DNA plays critical roles in regulations on nucleosome breathing.

In 1995, Polach, *et al*.^40^ first purposed nucleosome breathing, in which ends of nucleosomal DNA transiently unwrap and rebind histone protein. On single nucleosome level, nucleosome breathing exposes the binding site near entry/exit region to which transcription factors bind. It may also possible to enhance RNA polymerase binding to nucleosomal DNA. In addition, breathing might also increase the opportunity of histone exchange. Further studies suggested that nucleosome in chromatin fiber also underwent transient DNA breathing^41^. On chromatin level, breathing might change the compactness of chromatin fiber. More specifically, opening of the linker DNA might induce decompaction of chromatin fiber; closing of the linker DNA might induce compaction of chromatin fiber. Recent FRET studies reported that the linker DNA end-to-end distance was in the range of 4–8 nm^16^,^37^. The transition occurs on the order of 100 ms, which is beyond the capability of current MD simulations. However, with 10 independent simulations, we were able to observe the linker DNA opening and closing in our simulations, in which the DNA end-to-end distance varied from ~0 to ~8 nm. In addition, we analyzed the distance between the DNA sites to which fluorophores are attached in our simulation. The distance varied from ~2.5 to ~6.0 nm, covering the end-to-end distance range in breathing. More importantly, our study could provide molecular details of the structure even in the distance range of shorter than 4 nm, in which FRET experiments could not probe. Since the FRET efficiency strongly depends on the ratio of the donor–acceptor distance (D_0_) to the characteristic transfer distance, it lacks the spatial resolution to evaluate distances much shorter than D_0_. In most FRET studies, the dye pair used is FAM/TAMRA, Alexa 488/rhodamine-X, fluorescein/rhodamine-X or cy3/cy5^16^,^37^,^42^,^43^,^44^, with D_0_ in the range of 5–6 nm^42^,^43^,^44^,^45^,^46^. Therefore, in such studies, it is impossible to detect a distance of <2 nm or to report the closed conformation in principle. However, using all-atom simulations, we successfully observed the open and closed conformations and the pathway of closing process.

**Figure 7:**
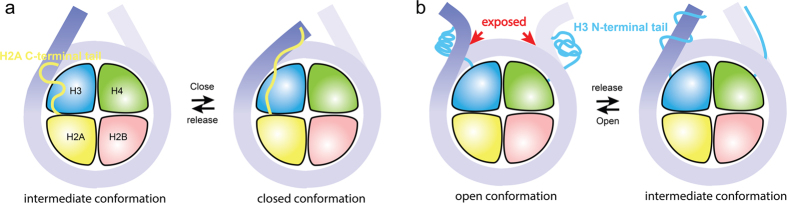
Model of linker DNA regulation by H2ACtT and H3NtT. (**a**) H2ACtT regulates the open and closed conformations of linker DNA by binding to different locations. The linker DNA is freely fluctuating when H2ACtT binds to the entry/exit region of DNA; whereas it is tightly constrained close to nucleosome core when H2ACtT binds to the distal end of linker DNA, resulting in the closed conformation of nucleosome. (**b**) H3NtT binds to the linker DNA in different patterns and regulates exposure of DNA at entry/exit region. H3NtTs without helix or compact coil conformations do not influence DNA conformation visibly. H3NtTs with helix or compact coil conformations tend to change the local curvature of DNA. The curvature changes potentially lead to exposure of the DNA at the entry/exit region to the solvent.

The correlation between the H2ACtT binding location and mobility of linker DNA suggest that tail binding to the distal end of DNA results in linker DNA moving close to the dyad axis, while binding to DNA at the entry/exit region allows linker DNA to move with less restriction (Fig. 3). Therefore, we speculated that H2ACtT governs the closing of nucleosome by moving along linker DNA as shown in Fig. 7a. The findings from H2ACtT truncated simulations support this mechanism (Figs 5 and 6). Ten independent simulations beginning with the intermediate conformation showed that the truncation of H2ACtT resulted in an increased DNA end-to-end distance (Fig. 5). Another two independent simulations beginning with the closed conformation showed that the truncation of H2ACtT destabilized the closed conformation and increased the opportunity for opening. These findings for H2ACtT’s role are consistent with previous findings on H2A variants and H2ACtT truncation studies^15^,^18^,^35^. H2A.Bbd, as an H2A variant lacking the C-terminal tail, has been found to activate gene transcription. A nucleosome with this variant has only 118 bp of DNA wrapped around it, indicating its partial unwrapping. In addition, the truncation of H2ACtT increased the rate of nucleosome repositioning on a DNA fragment^15^. In contrast, another H2A variant, MacroH2A, which has a longer C-terminal tail, is involved in transcriptional repression. Biochemical studies showed that the C-terminal non-histone fold region sterically blocks transcription factors and coactivators^47^,^48^.

In fact, the average DNA end-to-end distance did not change significantly by H3NtT truncation (Fig. 5). Biochemical study suggested H3NtT fast interacted with negatively charged DNA strands^49^. However, it was unclear whether the histone tails form a stable helix conformation or are in disordered conformations^22^,^50^,^51^,^52^. Recently, simulation studies have suggested α-helix formation in histone tails isolated from^51^ and in the presence of a nucleosome^53^. Our simulations showed the occurrence of both conformations in the presence of a nucleosome. Because H3NtT binds to the linker DNA in various patterns (Fig. 4), it regulates nucleosome dynamics in distinct ways. In most cases, the α-helix or compact coil of the histone tail approached and attached to the DNA from the outside, which causes the DNA curvature to change and potentially expose nucleosomal DNA at the entry/exit. On the other hand, histone tail wrapping around the linker DNA may increase the stiffness of DNA^54^,^55^, which reduces its fluctuation, possibly suppressing the linker DNA opening (Fig. 7b). Because of the various binding patterns of H3NtT, the correlation between H3NtT binding position and distance between the linker DNA end and dyad axis was not as clear as in the case of H2ACtT (Fig. 3, and Supplementary Figs S4 and S6). In addition, the distance was not affected by the truncation of H3NtT (Fig. 5). These results are consistent with previous studies using FRET experiment and MD simulation, in which truncation of the H3 tail did not significantly affect the linker DNA distance^16^,^35^.

Previous studies have shown that acetylation of the histone tail increases the propensity for an α-helix formation^50^,^53^. In addition, acetylation generally correlates with the activation of transcription, either through entry-site opening or destabilization of the nucleosome or chromatin^56^. Our results provide a possible explanation of the activation mechanism of acetylation. Acetylation induces α-helix formation, which may induce the linker DNA bending around it, exposing nucleosomal DNA. Meanwhile α-helix formation results in decreasing the propensity for H3NtT wrapping around linker DNA, avoiding strengthening DNA stiffness. Eventually, both effects would increase the possibility of nucleosomal DNA exposure.

In the closed conformation, H3NtT and H2ACtT both play pivotal roles. The simulation of opening process suggests that H3NtT is important for the stabilization of the closed conformation. H3NtT interacts with the linker DNA on both ends and forms a stable DNA–tail–DNA bridge (Supplementary Fig. S8), thereby maintaining the nucleosome in the closed conformation. The truncation of H3NtT disrupts the H3NtT-centered, DNA–tail–DNA bridge; thus, the nucleosome fluctuates between the closed and partially open states (Fig. 6). H2ACtT is also critical to the closed conformation. H2ACtT interacts with one linker DNA and the inner turn of DNA close to the dyad axis and forms another stable DNA–tail–DNA bridge (Supplementary Fig. S8), thereby it restricts linker DNA movement. Thus existing of either tails can stabilize nucleosome in closed conformation, but the truncation of both tails disrupts both DNA–tail–DNA bridges and induces the transition from a closed to an open conformation. This is clearly shown in Fig. 6 in which a considerable increase in fluctuation is observed when both are truncated compared with when one of the two is truncated.

H2ACtT and H3NtT are bridging the linker DNA and nucleosomal DNA, which is very similar to the role of linker histones in chromatosome. Linker histones interact with linker DNA and nucleosomal DNA as well to make chromatin compact^57^,^58^. And these interactions possibly restrain nucleosome dynamics^59^. Therefore the roles of H2AcT and H3NtT suggested in this study may be less pronounced in chromatosome or in the presence of linker histones. However, since H2ACtT is a targeting domain for the linker histone H1^18^, H2ACtT and H1 are inferred to cooperatively stabilize chromatin compaction.

In summary, our simulations provide insight into the closing and opening processes. We have shown the distinct roles of H3NtT and H2ACtT in nucleosome dynamics: H2ACtT simply restricts linker DNA mobility, whereas H3NtT regulates linker DNA in a complex way, which may enable the fine-tuning of nucleosome dynamics by the post-translational modification.

## Methods

### Initial nucleosome structure

Simulations were performed on intact nucleosome and on nucleosomes with truncation of H2ACtT, H3NtT, or both tails. The intact nucleosome structure in our study, which included eight histone proteins with all of the tails and 167 bp of DNA, was a hybrid of two nucleosome structures (PDB ID 1KX5 and PDB ID 1ZBB), as described in ref. 34. The tail truncation structures are all based on this hybrid structure. In terms of the truncated nucleosomes, for H2ACtT, the region from residue 118 to the C-terminal end was removed; for H3NtT, the truncation involved removal of the first 10 residues.

### Molecular dynamics simulations

The intact nucleosome was solvated with TIP3P water molecules in a rectangular box (17.6 × 15.4 × 10.5 nm). Crystallographic water molecules and ions were removed from the structure. Randomly chosen water molecules were substituted by Na^+^ and Cl^−^ ions to neutralize the system and to raise the system’s salt concentration to that in a physiological condition (150 mM). The system was then energy-minimized using the GROMACS^60^,^61^,^62^,^63^ package and the CHARMM27 force field^64^,^65^,^66^,^67^ for 20,000 steps of steepest descent and 50,000 steps of conjugate gradient algorithm until the maximum force was less than 10 kJ/mol/nm. Next, 10 different sets of initial velocity with an average temperature of 3 K were assigned to the minimized structure. Further, 10 annealing simulations were performed on the structure with assigned velocities, to obtain 10 independent initial structures for production simulations. Each annealing simulation lasted for 600 ps. The temperature was increased to 353 K at 1 K/ps for the first 350 ps and kept constant for the remaining 250 ps. The temperature was controlled by a V-rescale algorithm^68^. The time constant for the algorithm was set to 0.1 ps. In the following 1-ns simulation, with the temperature being controlled, the pressure was maintained at 1.0 atm using a Parrinello–Rahman algorithm^69^,^70^ with a time constant of 2.0 ps. Finally, 10 different initial structures were obtained for the production runs.

Each production simulation lasted for 100 ns. The production simulations were performed at 1 atm and 353 K. Periodic boundary conditions were used. Electrostatic interactions were calculated by the Particle Mesh Ewald method^71^, with maximum spacing at 0.16 nm and cubic interpolation. Van der Waals interactions were cut at 1.2 nm with switching^72^ between 1.0 and 1.2 nm. Bonds with hydrogen were constrained by a LINCS algorithm. The accuracy, namely, lincs order, and iterative correction, namely, lincs iteration, were set to 4 and 1, respectively.

Using the same strategy as that for the abovementioned intact nucleosome system, two different initial structures for each of the systems with either H2ACtT or H3NtT truncated were obtained. For each structure, a 60-ns long production simulation was performed. In addition, the closed conformation obtained from the production simulation of the intact nucleosome was then used as the starting conformation in the opening simulation. Moreover, the closed structure was modified by truncating H2ACtT, H3NtT, or both tails, as shown in Fig. 5a. For all four structures, two independent initial structures were obtained by distinct relaxation simulations on the closed conformation. Eventually, two free molecular dynamic simulations were performed for each. All simulations conducted in this study are listed in Table 1.

### The contact between Histone tail and DNA

The interactions between histone tails and DNA were characterized by contact position numbers. The smaller the position number becomes the more distal position of DNA the histone tail contacts. The contact was defined by the distance between atoms on histone tail and DNA base pair. If the minimum distance between these two groups of atoms was less than 0.6 nm, we considered the histone tail contacted with this specific base pair. The smallest number of base pair, to which histone tail contacted, was recorded as the contact position.

### The H3NtT conformation analysis

Atomic coordinates of H3NtT saved every 100 ps were used for conformational analysis. Secondary structure was analyzed with DSSP^73^,^74^. The total count of helix structure (including α-helix, 3–10 helix, and π-helix in DSSP) of each amino acid during the entire simulation was recorded and plotted in Supplementary Fig. S7. If a helix forms over half of the simulation time, we consider it tail formed a stable helix. The RG of the first 37 amino acid of H3NtT was also calculated using the last 80 ns in each simulation run. The average and 95% confidence interval of RG with helix was calculated. The tail with RG less than this lower limit of 95% confidence interval was considered as compact conformation. Therefore, tail *A* in simulation 1 and tail *B* in simulation 3 were considered as compact coil (Supplementary Fig. S7).

### Local bending angle analysis

The local bending angle was defined as shown in Supplementary Fig. S7. In the analysis, five consecutive base pairs were treated as one segment. The directions of four base pair steps in one segment were calculated by the program 3DNA^75^. The local helix axis direction was obtained by the averaging these four directions. Local bending angle was calculated by two DNA segments with a gap of two consecutive base pair steps.

## Additional Information

**How to cite this article**: Li, Z. and Kono, H. Distinct Roles of Histone H3 and H2A Tails in Nucleosome Stability. *Sci. Rep.* **6**, 31437; doi: 10.1038/srep31437 (2016).

## Supplementary Material

Supplementary Information

Supplementary Movie S1

Supplementary Movie S2

Supplementary Movie S3

## Figures and Tables

**Table 1:** MD simulations carried out in this study.

Nucleosome	Intact	DH2A	DH3	DH3′	DH2A+DH3
Free MD	10 × 100 ns	10 × 60 ns	10 × 60 ns	6 × 60 ns	—
Opening*	2 × 100 ns	2 × 100 ns	2 × 100 ns	—	2 × 100 ns

^*^MD started with a closed conformation.
