# Supplementary material for: Distinct Roles of Histone H3 and H2A Tails in Nucleosome Stability
Source: Sci Rep. 2016 Aug 16;6:31437. doi: 10.1038/srep31437 (PMC4985630; doi:10.1038/srep31437)
Supplement: Supplementary Information [file srep31437-s1.pdf]

Supplementary material

Distinct Roles of Histone H3 and H2A Tails in Nucleosome Stability

Zhenhai Li, and Hidetoshi Kono

Molecular Modeling and Simulation Group

National Institutes for Quantum and Radiological Science and Technology

8-1-7 Umemidai, Kizugawa, Kyoto 619-0215, Japan

Supplementary material includes 3 videos and 10 supplementary figures.

Supplementary Video 1. Nucleosome opening. The video shows the simulation trajectory of nucleosome opening process. H2ACtT has not reached the distal ends of linker DNA, thus lacking constraint to the linker DNA.

Supplementary Video 2. Nucleosome closing. The video shows the simulation trajectory of nucleosome closing process. Both H2ACtT and H3NtT play pivotal roles in the closing process.

Supplementary Video 3. H2ACtT function in closing process. Zoom in the interaction between H2ACtT and linker DNA. The video shows H2ACtT jumping between the linker DNA and the inner loop of nucleosomal DNA, and restrict the linker DNA movement after stably binding to both DNA segments. H2ACtT and DNA strand within 0.5 nm of H2ACtT are highlighted with a bead-stick model.

Supplementary Figure S1

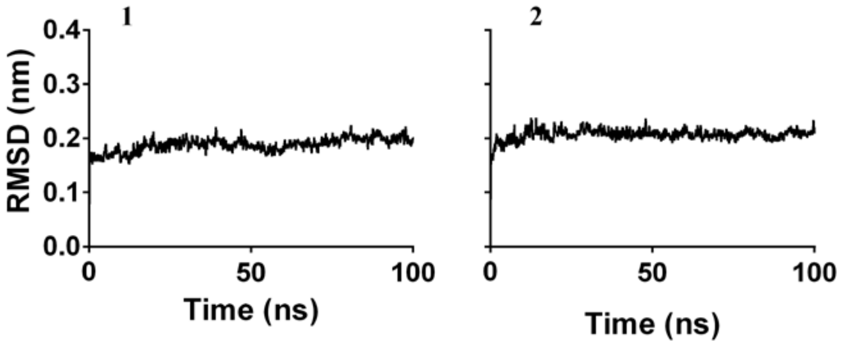

Supplementary Figure S1. RMSD of simulations at 300 K. The RMSD was calculated for the histone fold domains (H3: residue 45-135; H4: residue 27-102; H2A: residue 17-104; H2B: 36-122) and the central 127 bp segment of nucleosomal DNA. Left and right panels are RMSD for two independent simulations at 300 K.

Supplementary Figure S2

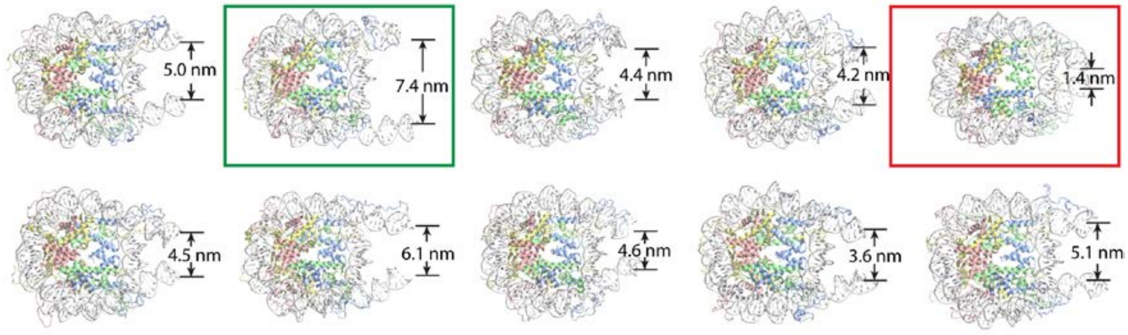

Supplementary Figure S2. Snapshots of the last frames in 10 independent simulations. Top view of the last frames of 10 independent simulations. DNA and histone protein are denoted in different colors. The color codes are the same as Fig. 1. The distances of linker DNA end in the particular frames are marked in the figures. The second and fifth simulations were highlighted by green and red boxes, showing the opened and closed conformation.

Supplementary Figure S3

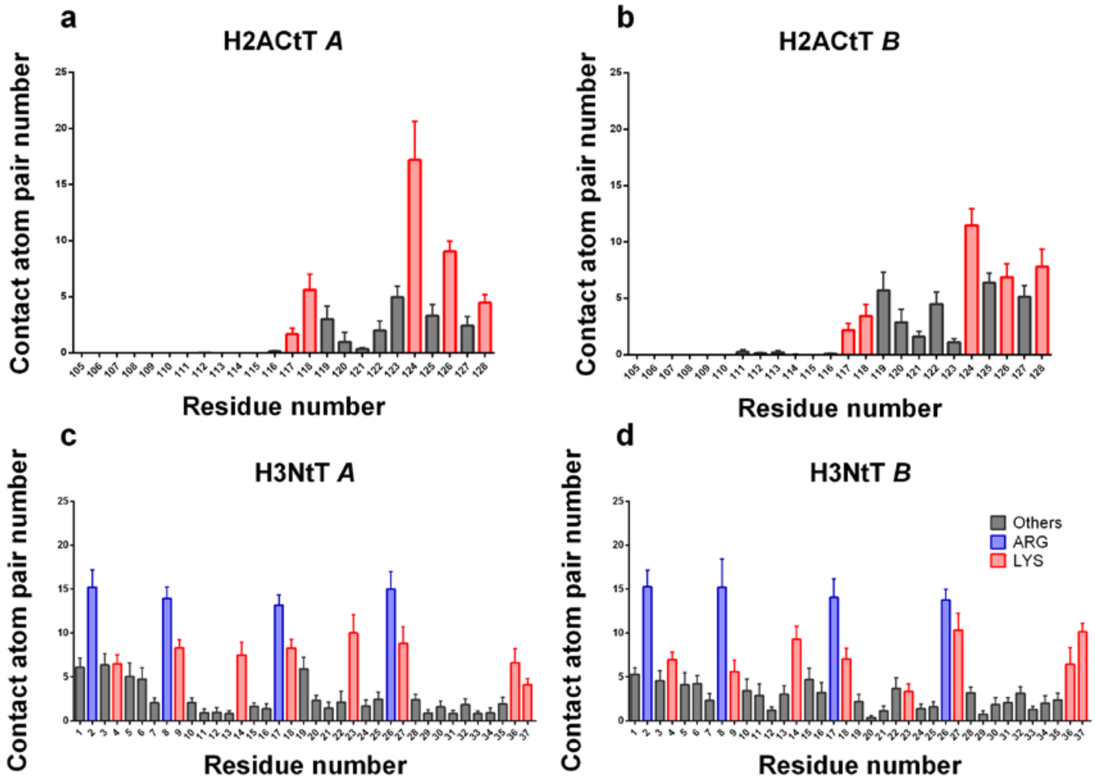

Supplementary Figure S3. Average number of contact atom pairs between tails and DNA. Indices "*A*" and "*B*" indicate two ends of the linker DNA. *a, b.* show the average number of contacts of each amino acid on H2ACtT copy *A* (a) and copy *B* (b), respectively. *c, d.* show the average number of contacts of each amino acid on H3NtT copy *A* (c) and copy *B* (d). Contact is counted when distance of a pair of atoms is less than or equal to 3Å. Standard errors in the figures are calculated from the independent contact numbers obtained from different simulation runs.

Supplementary Figure S4

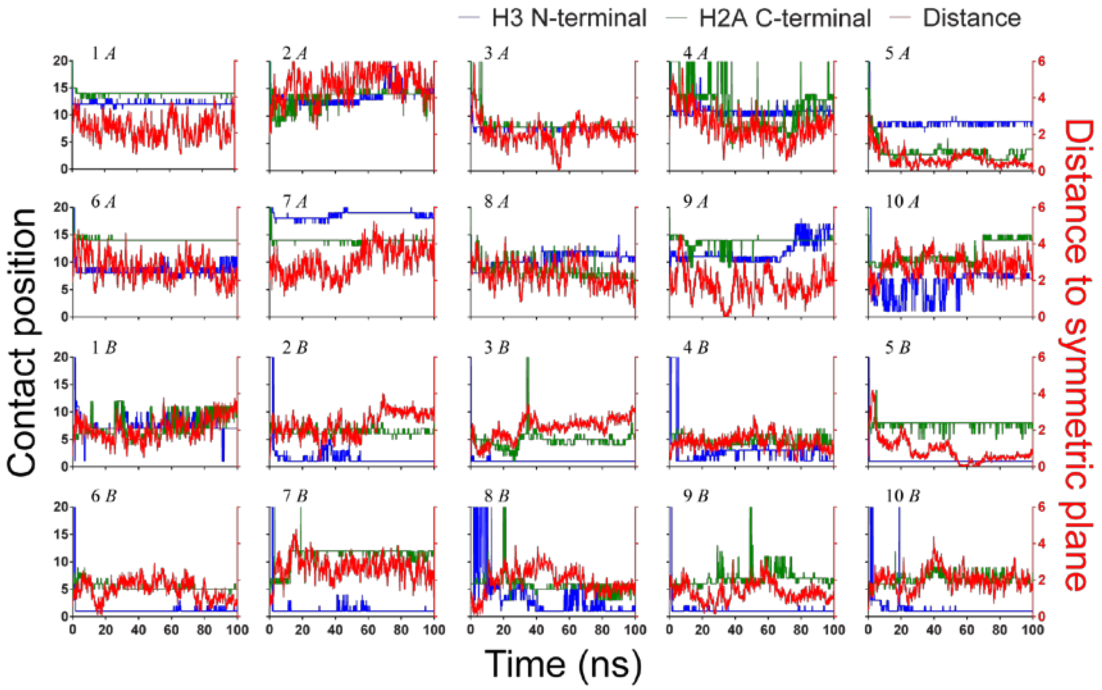

Supplementary Figure S4. The correlation between the contact position by tails and the linker DNA mobility. The variation of histone tail H3NtT or H2ACtT contacting DNA position along with the time were plotted as blue or green curves, respectively. The distances from the linker DNA end to the symmetric plane were plotted as red curves as well. Each panel shows one side of linker DNA and corresponding histone tails from a single simulation. The number in top left corner of each panel shows the simulation number, and indices "*A*" and "*B*" indicate two sides of the linker DNA respectively.

Supplementary Figure S5

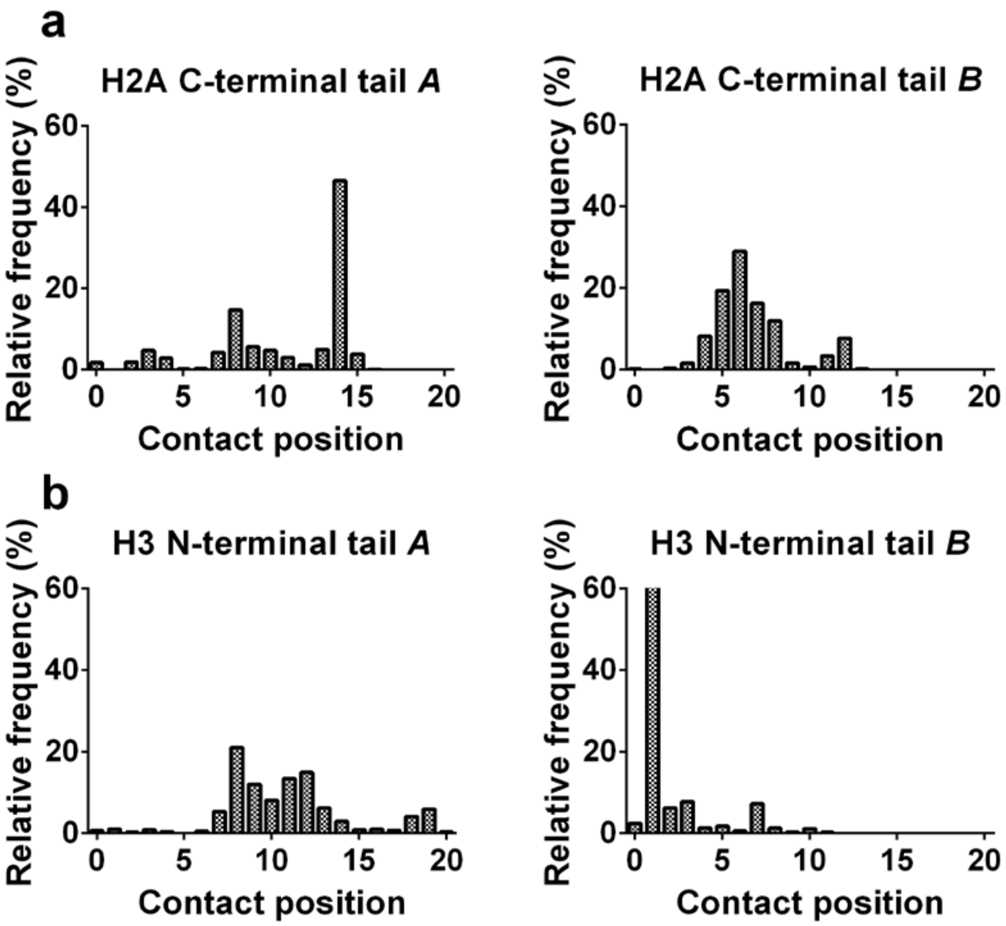

Supplementary Figure S5. Distributions of the most distal DNA position contacted by H2ACtT and H3NtT. *a.* Distribution of the most distal DNA position contacted by H2ACtT in all the frames of 10 independent simulation. *b.* Distribution of the most distal DNA position contacted by H3NtT in all the frames of 10 independent simulation. Indices “*A*” and “*B*” indicate two ends of the linker DNA.

Supplementary Figure S6

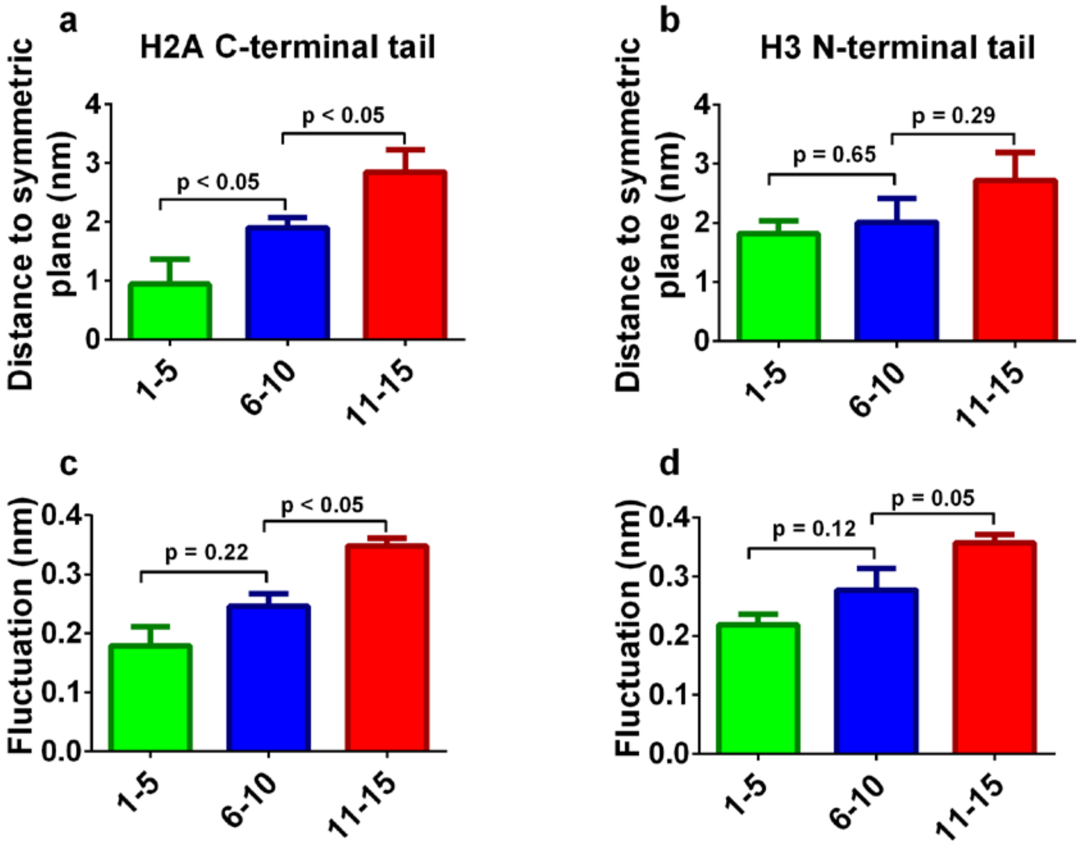

Supplementary Figure S6. The correlation between the most distal DNA position contacted by tails and the inker DNA motility. *a, c.* show the correlation between H2ACtT-DNA contact region and the linker DNA motility. *b, d.* show the correlation between H3NtT-DNA contact region and linker DNA motility. *a, c.* show that the distances vary with the DNA region histone tails contact. *c, d.* show that the distance fluctuations vary with the DNA region histone tails contact. We calculated the standard deviation of the distance within 1 ns to characterize this fluctuation. Both ends of DNA were considered together. Standard errors in the figures are calculated from the independent distances or fluctuations obtained from different simulation runs. The P-value is calculated by two-tailed t-test.

Supplementary Figure S7

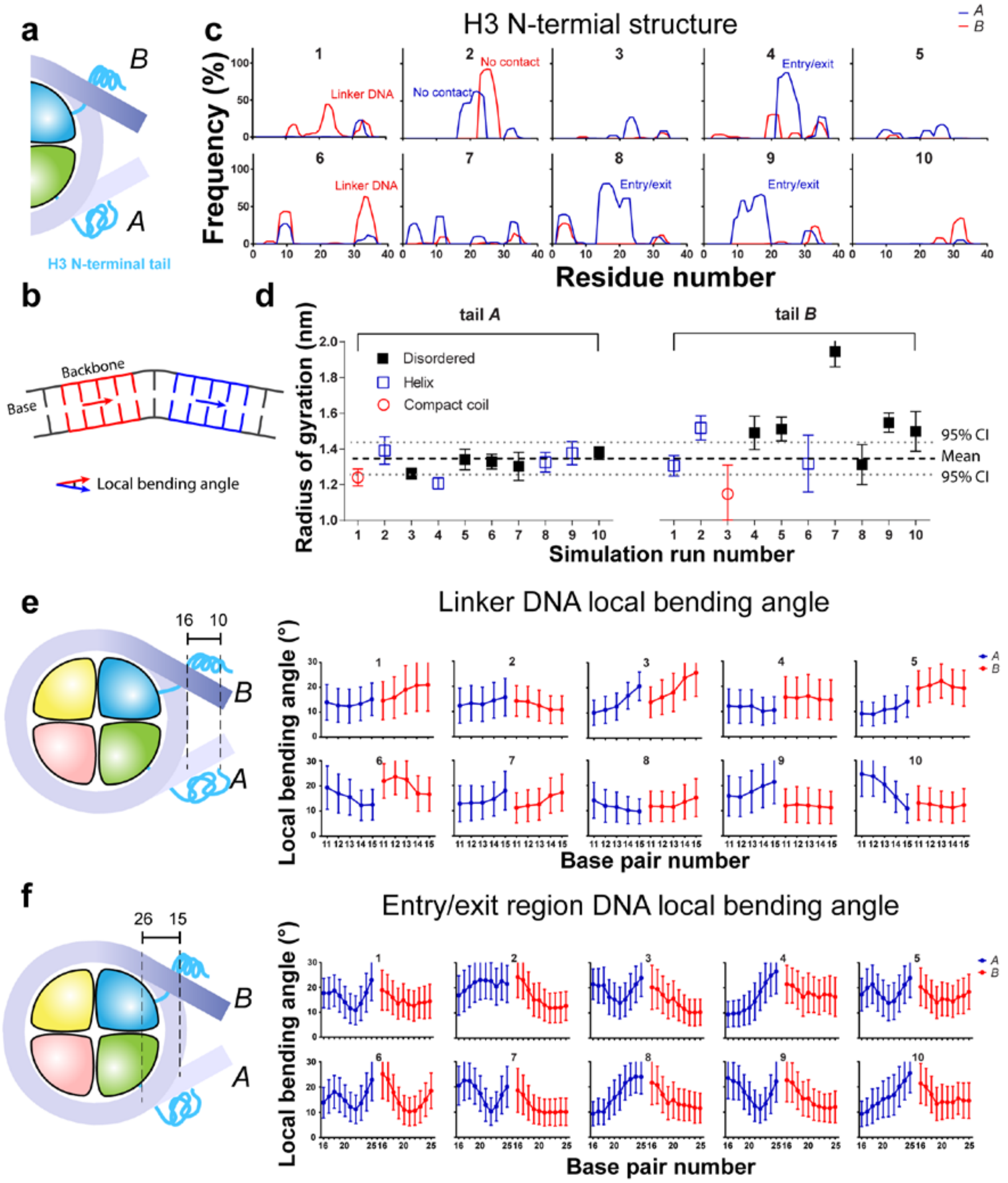

Supplementary Figure S7. H3 N-terminal structure and DNA bending angle analysis. *a*. Schematic of nucleosome particle. Indices "*A*" and "*B*" distinguish two H3NtTs. *b*. Definition of local bending angle in DNA strands. Two axis vectors are calculated for two DNA segments as

highlighted with red and blue colors. The local bending angle is defined by the angle between these two vectors. *c*. The total count of helix structure formed on each amino acid in two H3NtTs (*A* and *B* as indicated in (a)) during the ten simulation runs. The simulation run number was listed on top of each panel. The locations helices contacts with DNA are indicated in the figures. *d.* The average of H3NtT RGs within the last 80 ns for each of ten simulation runs. The tail with different secondary structures was labeled with different colors as indicated in the legends. The black dash and grey dot lines indicate the average RG and upper/lower limit of 95% confidence interval of H3NtT with helix. *e.* The average and standard deviation (SD) of DNA local bending angles from base pair 10 to 16 on both DNA ends (denoted by *a* in blue and *b* in red) in each simulation runs. Base pair number is the number of the middle base pair as shown in (b). The simulation run number was listed on top of each panel. *f*. The average and SD of DNA local bending angles from base pair 15 to 26 on both ends (denoted by *a* in blue and *b* in red) in each simulation run. Base pair is numbered in the same way as (e). The simulation run number was listed on top of each panel. The error bars in *d*, *e*, and *f* indicate standard deviation.

Supplementary Figure S8

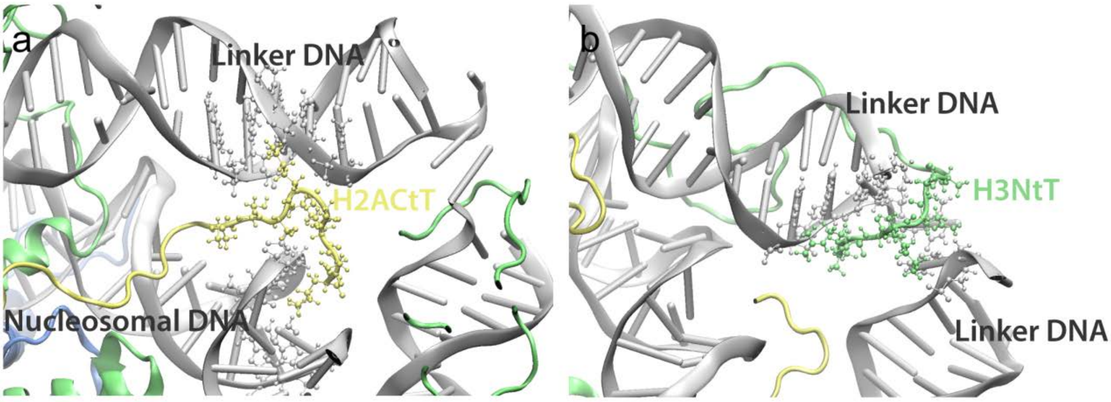

Supplementary Figure S8. DNA-tail-DNA bridges. *a.* A DNA-tail-DNA bridge formed by the linker DNA, H2ACtT and the inner loop of nucleosomal DNA are shown. *b.* A DNA-tail-DNA bridge formed by the two linker DNAs and H3NtT are shown.

Supplementary Figure S9

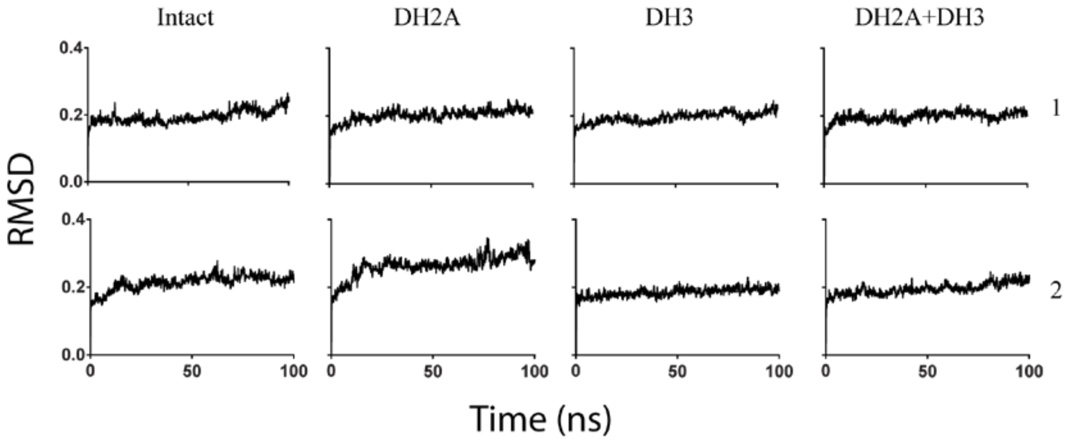

Supplementary Figure S9. The RMSD of reopening simulation. The RMSD of reopening process of intact, H2ACtT truncated (DH2A), H3NtT truncated (DH3), and both H2ACtT and H3NtT truncated (DH2A+DH3) nucleosomes in eight independent simulation runs (2 simulation runs for each condition). The RMSD was calculated for histone fold domains (H3: residue 45-135; H4: residue 27-102; H2A: residue 17-104; H2B: 36-122) and the centeral 127 bp segment of nucleosomal DNA. Upper and lower panels show two independent simulation results.

Supplementary Figure S10

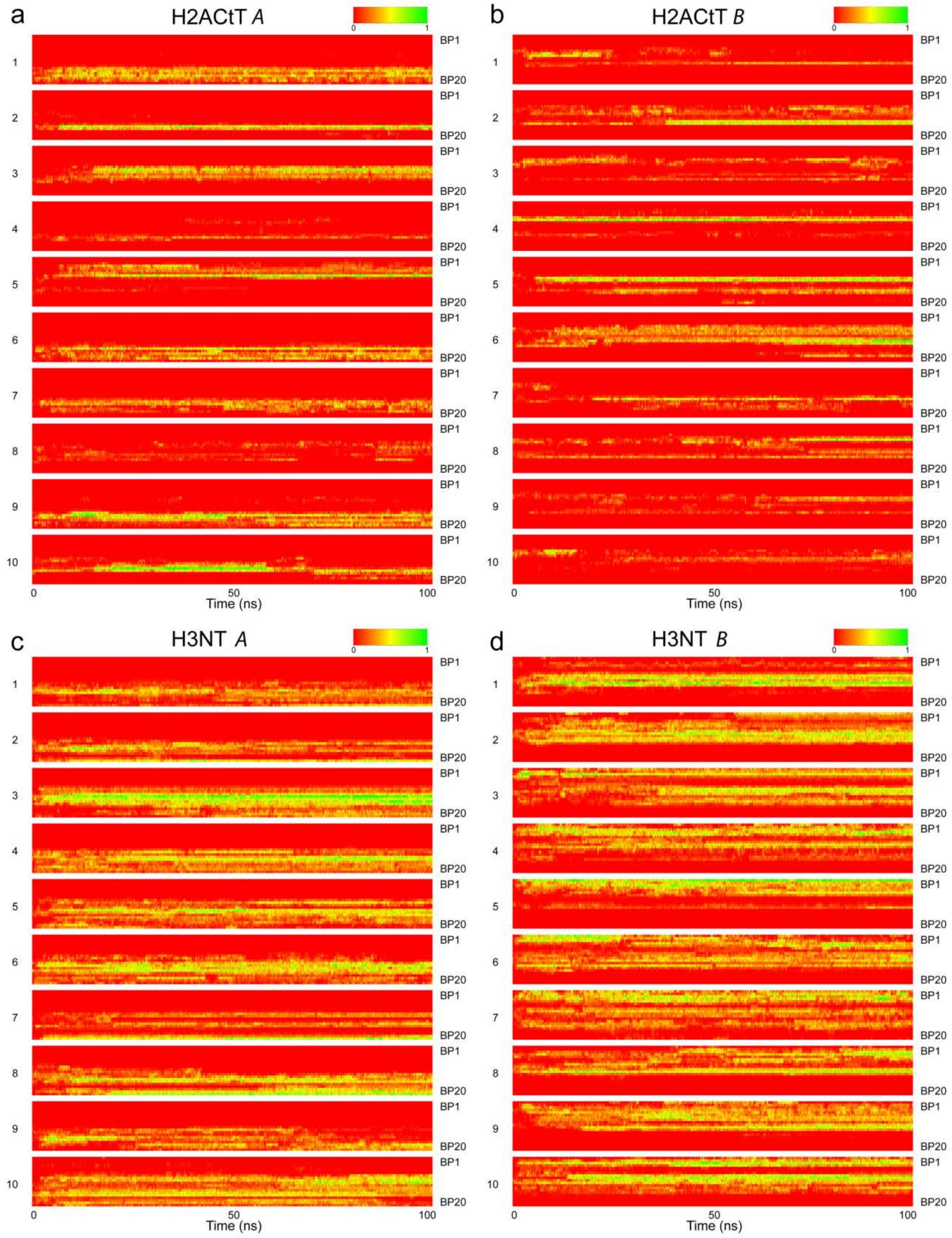

Supplementary Figure S10. Time trace of histone tail-DNA contact. Figures show the normalized contact number between H2ACtT (normalized by 20) and each DNA base pair variation along time (a and b) and the normalized contact number between H3NtT (normalized

by 30) and each DNA base pair. (c and d). The contact numbers have the same definition as in Fig. S3. *a, c.* denote copy "*A*" of the tails. *b, d.* denote copy "*B*" of the tails. In each sub-figure, 10 panels represent 10 independent simulation runs. Contact by tails on base pair 1 (BP1) to base pair 20 (BP20) (starting from the DNA end) were analyzed and plotted. Color change in each line of the panel indicates the normalized contact number varies along the time. One can observe that the location where DNA base pairs make most contact with histone tail does not change significantly within 100 ns.
